# CARETestLung: A mechanical test lung with Configurable airway Resistance, lung Elastance, and breathing efforts

**DOI:** 10.1016/j.ohx.2024.e00579

**Published:** 2024-08-28

**Authors:** Wei Yang Tay, Christopher Yew Shuen Ang, Yeong Shiong Chiew, J. Geoffrey Chase

**Affiliations:** a Monash University Malaysia, Jalan Lagoon Selatan, Bandar Sunway, 47500 Subang Jaya, Selangor, Malaysia; b Department of Mechanical Engineering, University of Canterbury, Christchurch 8041, New Zealand

**Keywords:** Mechanical ventilation, Mechanical test lung, Spontaneous breathing

## Abstract

A mechanical test lung is a crucial tool in accurately simulating patient-specific physiological responses of patients undergoing mechanical ventilation (MV), which, in turn, offer clinicians insight into lung mechanics during MV. In particular, it can be used to facilitate better methods to identify optimal ventilator settings, modes for individual patients by providing a platform to experiment with different MV settings. This addresses the challenge of optimising MV settings caused by variability in pathological conditions and the progression of respiratory disease over time within patients. However, the accessibility and cost of versatile test lungs limit widespread adoption in clinical settings, underscoring the need for affordable alternatives. This paper presents detailed instructions for the design and construction of a replicable, cost-effective mechanical test lung. The design features 3 subsystems: 1) the lung compartment; 2) the airway; and 3) a spontaneous breathing system. A detailed tests series shows its ability to replicate clinically realistic lung elastance values ranging from 25 to 85 cmH_2_O/L and airway resistance values from 10 to 45 cmH_2_O·s/L. It can also simulate a range of clinically realistic spontaneous breathing patterns. These capabilities yield pressure and flow ventilation data comparable to certified clinical test lungs across diverse scenarios, as well as matching clinically observed behaviours and dynamics. This accessible and versatile test lung offers valuable opportunities for optimising MV settings and advancing patient care, as well as its use in developing a range of physiological models for model-based decision support.

## Graphical abstract

**Figure:**
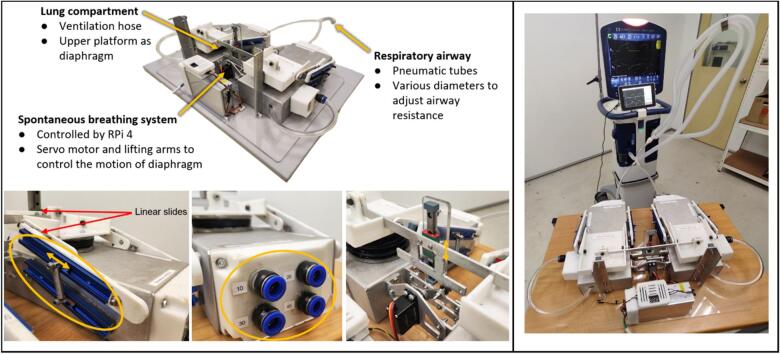

**Specifications table**.

**Table:** 

Hardware name	CARE Test Lung (Mechanical Test Lung with Configurable Airway Resistance, Lung Elastance, and Breathing Efforts)
Subject area	• Engineering and materials science • Medical (e.g., pharmaceutical science) • Educational tools and open-source alternatives to existing infrastructure
Hardware type	• Mechanical engineering and materials science • Mechanical test lung
Closest commercial analog	Michigan Dual Adult Lung Simulator
Open source license	Creative Commons Attribution-ShareAlike 4.0
Cost of hardware	∼$ 730 USD
Source file repository	Mechanical Test Lung - Mendeley Data01Mechanical Test Lung - Mendeley Data02

## 1 Hardware in context

Mechanical ventilation (MV) is the primary treatment for patients with respiratory failure. It aims to ensure sufficient oxygenation in patients while avoiding ventilator-induced lung injury (VILI) [1]. Nevertheless, the guidelines available from standard clinical protocols for optimising MV settings are restricted, despite extensive research over decades [2]. This limitation arises due to the variability in pathological conditions and the progression of respiratory disease over time within an individual patient. This variability is not only between patients but also within the same patient, given the heterogeneous nature of a patient's lung suffering from respiratory failure [2]. What works for one patient in terms of MV settings might lead to VILI in another [2]. Conversely, settings that recruit a specific lung region might cause distention in another area [3], [4]. Consequently, universally applicable MV settings for every patient do not exist. As the optimal MV settings are patient-specific, the success of the treatment heavily relies on the experience and intuition of the clinician [2], [5]. Therefore, more effective approaches for determining patient specific MV settings are imperative to enhance outcomes for everyone.

Utilising a mechanical test lung to simulate the respiratory process is one of the existing solutions, offering a means to visually understand the physiological response of the human lung during ventilation by replicating its fundamental mechanics [3], [6], [7]. Consequently, having a mechanical test lung equipped with reasonable specifications and adjustable parameters like lung elastance and airway resistance becomes instrumental in representing the complexities of the patient’s lung in the context of MV therapy. This setup facilitates the exploration of various MV settings tailored to specific lung conditions, enabling the observation of outcomes and aiding in the identification of optimal settings for individual patients.

However, the current challenge lies in the accessibility and cost of test lung models with such functionalities, making it crucial to design and develop a more affordable and accessible mechanical test lung. The test lung envisioned should possess adjustable lung mechanics, focusing on fundamental lung mechanics parameters such as lung elastance and airway resistance. It should also be capable of simulating spontaneous patient breathing efforts encountered during MV treatment under assisted spontaneous breathing MV modes, which are increasingly commonly used in care, as they require less sedation [2].

This paper details the development of a mechanical test lung with two independent lung compartments and a spontaneous breathing mechanism that is designed based on the linear single-compartment model. Specifically, the mechanical test lung is named CARE Test Lung (Mechanical Test Lung with Configurable Airway Resistance, Lung Elastance and Breathing Efforts). Although the single-compartment model cannot fully capture the complexity and non-linearity of the human respiratory system, it remains the most clinically feasible solution as it requires fewer clinical protocols and invasive measurements [8]. Moreover, simpler models benefit from fewer trade-offs between parameters, which can lead to more accurate parameter estimation and predictions [9]. Compared to complex models, this advantage is particularly significant in situations with limited resources, enhancing the model's reliability and practicality in clinical settings.

The test lung features an active control system for emulating the spontaneous breathing of various patients. With adjustable lung elastance and airway resistance, the model effectively serves its core purpose of replicating diverse respiratory system characteristics. The standalone control system, utilising Raspberry Pi (RPi), facilitates incorporation of a clinically realistic range of breathing patterns into the test lung's spontaneous breathing simulation. Thus, the proposed test lung opens avenues for further exploration into patient-specific spontaneous breathing efforts, but in a replicable manner not available from clinical data. These refinements and investigations collectively contribute to an enhanced comprehension of human respiratory mechanics, including the more rapid development of virtual patient or digital twin models to guide care, paving the way for advanced and well-informed respiratory care solutions [5], [10], [11]. Furthermore, it helps to narrow the educational gap in clinical training in developing countries by offering an accessible test lung with low material costs and a simple fabrication process.

## 2 Hardware description

**Fig. 1:**
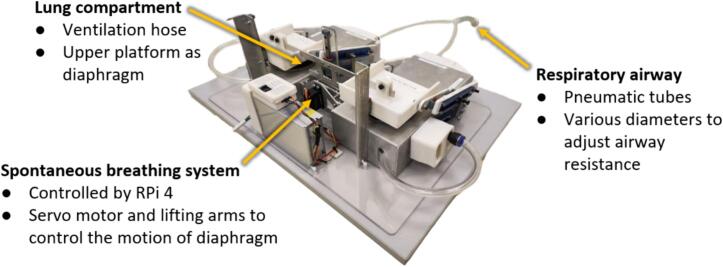
Major subsystems in the CARE Test Lung.

The featured test lung in this paper comprises three key subsystems illustrated in Fig. 1, including the lung compartment, respiratory airway, and spontaneous breathing system. With two individual lung compartments, each integrating a spring-based mechanism for adaptable lung elastance, the test lung can capture the heterogeneous characteristics of a patient's respiratory system. The respiratory airway, which is constructed with a network of pneumatic tubes, enables an adjustable airway resistance. Meanwhile, the spontaneous breathing system employs an RPi 4 Model B and a 9imod DSC45MG servo motor to emulate human respiratory efforts, producing diverse breathing patterns.

**Fig. 2:**
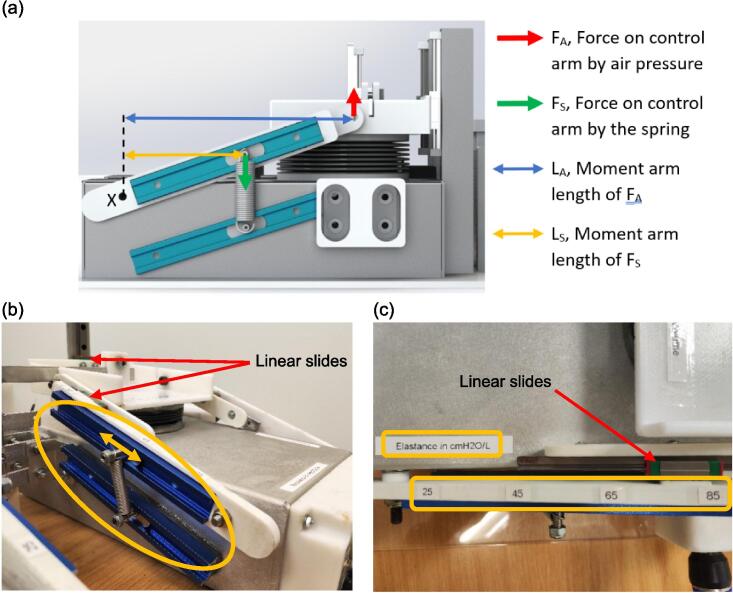
(a) Spring mechanism for adjusting lung elastance (b) Lung elastance can be adjusted by changing the position of the spring along the aluminium tracks (c) Lung elastance settings (25 – 85 cmH_2_O/L).

**Fig. 3:**
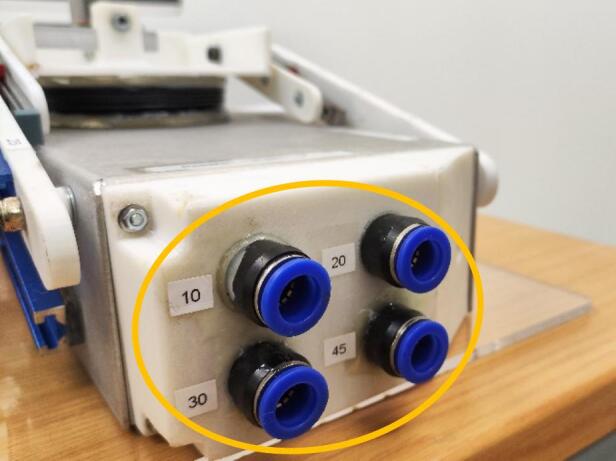
Respiratory airways with different resistance settings (10, 20, 30 or 45 cmH_2_O·s/L).

The mechanism enabling the continuous adjustment of lung elastance is depicted in Fig. 2(a). This design concept involves manipulating the moment arm length of the spring force, L_S_, while keeping that of the air pressure force, L_A_, fixed. The lower arm is fixed, whereas the upper arm is only fixed at point X. Assuming a zero resultant moment about a pivoting point X, a greater L_S_ leads to a larger F_A_, subsequently raising the lung elastance. Through this mechanism in Fig. 2(b), lung elastance can be fine-tuned to the desired value by positioning the spring based on the calibration illustrated in Fig. 2(c). Meanwhile, as shown in Fig. 3, there are four parallel airways built into each lung compartment of the test lung, each with a different length and diameter to allow variation in the airway resistance. The respiratory airway can be further extended with pressure and flow sensors [12] for monitoring of the airway waveform at the tracheal position of the test lung.

**Fig. 4:**
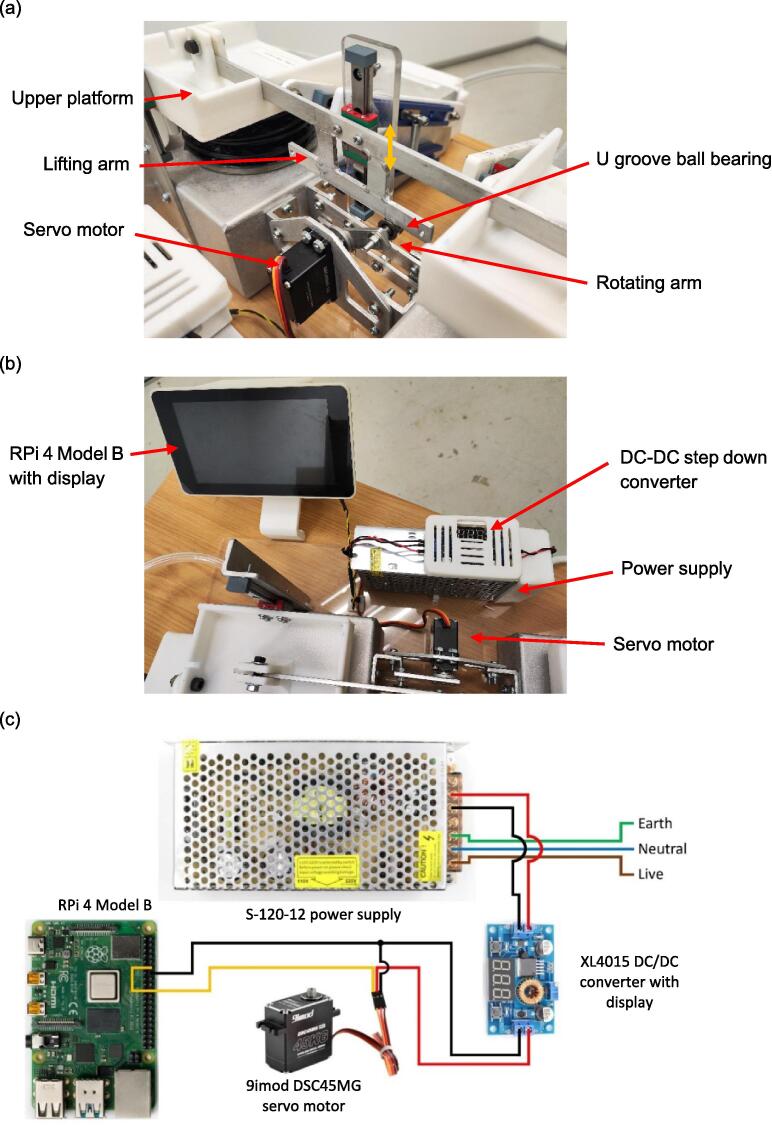
(a) Servo motor and the accessory lifting arms that controls the motion of the diaphragm (b) RPi 4 Model B with display that controls the servo, along with the accessory electronics (c) Circuit diagram of the spontaneous breathing system.

The spontaneous breathing system replicates the diaphragm's contraction and relaxation by controlling the vertical movement of the lung compartment's upper platform. This control involves converting the servo motor's rotational motion to the linear motion of the upper platform via the mechanism in Fig. 4(a), showing a U-groove ball bearing affixed to the end of a rotating arm connected to the servo motor's output shaft. As the servo motor's output shaft rotates, the bearing on the rotating arm elevates the lifting arm, in turn raising the upper platform of both lung compartments. Fig. 4(b) illustrates the controller and other electronic components and Fig. 4(c) shows the wiring connection for the spontaneous breathing system.

## 3 Design files summary

The repository contains the SOLIDWORKS Part files for all custom components, whereas the master model named “Mechanical test lung”, which contains all components and their interconnections, is provided as a SOLIDWORKS Assembly file. Additionally, STL files for 3D printed parts are included, along with SOLIDWORKS Drawing files for components not suitable for 3D printing. Among the non-3D printable parts, the DXF files are also provided for parts suitable for laser cutting.

**Table:** 

**Design file name**	**File type**	**Open source license**	**Location of the file**
Mechanical test lung	SLDASM	CC BY 4.0	Folder: ‘Hardware’
Aluminium T track	SLDPRT	CC BY 4.0	Folder: ‘Hardware’
Aluminium base	SLDPRT	CC BY 4.0	Folder: ‘Hardware’
Aluminium base mirrored	SLDPRT	CC BY 4.0	Folder: ‘Hardware’
Connectors holder part 1	SLDPRT	CC BY 4.0	Folder: ‘Hardware’
Connectors holder part 2	SLDPRT	CC BY 4.0	Folder: ‘Hardware’
Control arm A	SLDPRT	CC BY 4.0	Folder: ‘Hardware’
Control arm B	SLDPRT	CC BY 4.0	Folder: ‘Hardware’
Custom rod	SLDPRT	CC BY 4.0	Folder: ‘Hardware’
Flat bar A	SLDPRT	CC BY 4.0	Folder: ‘Hardware’
Flat bar B	SLDPRT	CC BY 4.0	Folder: ‘Hardware’
Flat bar C	SLDPRT	CC BY 4.0	Folder: ‘Hardware’
Hose base	SLDPRT	CC BY 4.0	Folder: ‘Hardware’
Hose cover	SLDPRT	CC BY 4.0	Folder: ‘Hardware’
L bracket	SLDPRT	CC BY 4.0	Folder: ‘Hardware’
Linear guide rail stopper	SLDPRT	CC BY 4.0	Folder: ‘Hardware’
Linear rail mount A	SLDPRT	CC BY 4.0	Folder: ‘Hardware’
Linear rail mount B	SLDPRT	CC BY 4.0	Folder: ‘Hardware’
PVC aluminium ducting hose	SLDPRT	CC BY 4.0	Folder: ‘Hardware’
Power supply cover	SLDPRT	CC BY 4.0	Folder: ‘Hardware’
Servo motor mount	SLDPRT	CC BY 4.0	Folder: ‘Hardware’
Spacer	SLDPRT	CC BY 4.0	Folder: ‘Hardware’
Tubes cover	SLDPRT	CC BY 4.0	Folder: ‘Hardware’
Tubes holder	SLDPRT	CC BY 4.0	Folder: ‘Hardware’
Upper platform	SLDPRT	CC BY 4.0	Folder: ‘Hardware’
Ventilator adapter	SLDPRT	CC BY 4.0	Folder: ‘Hardware’
Voltage regulator cover	SLDPRT	CC BY 4.0	Folder: ‘Hardware’
Voltage regulator holder	SLDPRT	CC BY 4.0	Folder: ‘Hardware’
Aluminium base	SLDDRW	CC BY 4.0	Folder: ‘Hardware’
Custom rod	SLDDRW	CC BY 4.0	Folder: ‘Hardware’
Flat bar A	SLDDRW	CC BY 4.0	Folder: ‘Hardware’
Flat bar B	SLDDRW	CC BY 4.0	Folder: ‘Hardware’
Flat bar C	SLDDRW	CC BY 4.0	Folder: ‘Hardware’
L bracket	SLDDRW	CC BY 4.0	Folder: ‘Hardware’
Linear rail mount A	SLDDRW	CC BY 4.0	Folder: ‘Hardware’
Connectors holder part 1 (mirrored)	STL	CC BY 4.0	Folder: ‘3D files’
Connectors holder part 1	STL	CC BY 4.0	Folder: ‘3D files’
Connectors holder part 2	STL	CC BY 4.0	Folder: ‘3D files’
Control arm A	STL	CC BY 4.0	Folder: ‘3D files’
Control arm B	STL	CC BY 4.0	Folder: ‘3D files’
Linear guide rail stopper	STL	CC BY 4.0	Folder: ‘3D files’
Power supply cover	STL	CC BY 4.0	Folder: ‘3D files’
Tubes cover	STL	CC BY 4.0	Folder: ‘3D files’
Upper platform	STL	CC BY 4.0	Folder: ‘3D files’
Ventilator adapter	STL	CC BY 4.0	Folder: ‘3D files’
Voltage regulator cover	STL	CC BY 4.0	Folder: ‘3D files’
Voltage regulator holder	STL	CC BY 4.0	Folder: ‘3D files’
Flat pattern – Aluminium base	DXF	CC BY 4.0	Folder: ‘2D files’
Flat pattern – Aluminium base_mirrored	DXF	CC BY 4.0	Folder: ‘2D files’
Hose base	DXF	CC BY 4.0	Folder: ‘2D files’
Hose cover	DXF	CC BY 4.0	Folder: ‘2D files’
Linear rail mount B	DXF	CC BY 4.0	Folder: ‘2D files’
Servo motor mount	DXF	CC BY 4.0	Folder: ‘2D files’
Spacer	DXF	CC BY 4.0	Folder: ‘2D files’
Tubes holder	DXF	CC BY 4.0	Folder: ‘2D files’
ramp_fq10	py	CC BY 4.0	Folder: ‘RPi Code’
ramp_fq20	py	CC BY 4.0	Folder: ‘RPi Code’
sin_fq10	py	CC BY 4.0	Folder: ‘RPi Code’
sin_fq20	py	CC BY 4.0	Folder: ‘RPi Code’

The repository can be accessed through the following link:

Mechanical Test Lung - Mendeley Data01.

## 4 Bill of materials summary

The cost of materials for the CARE Test Lung is USD$ 729.67, and the bill of materials can be found at: Mechanical Test Lung - Mendeley Data02.

## 5 Build instructions

### 5.1 Lung compartment

**Fig. 5:**
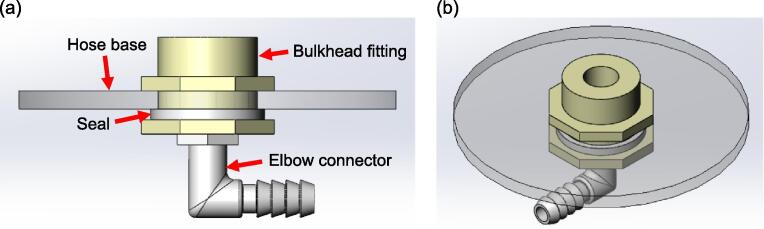
(a) Side view of the hose base assembly (b) Isometric view of the hose base assembly.

**Fig. 6:**
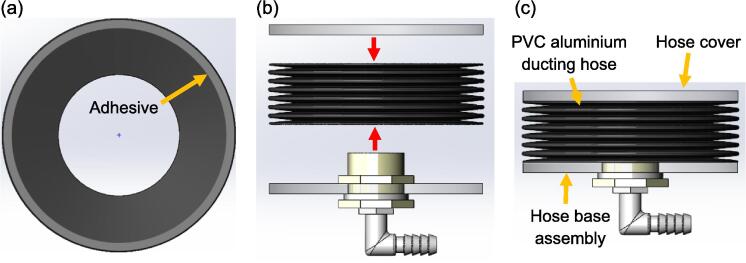
Steps to fabricate the lung compartment: (a) Apply adhesive to the edge of the ducting hose’s top and bottom surfaces (b) Attach the hose cover and hose base assembly to the ducting hose (c) Side view of the lung compartment.

**Fig. 7:**
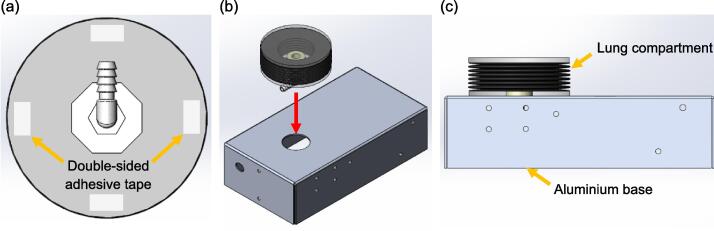
Steps to attach the lung compartment on the aluminium base: (a) Attach four double sided adhesive tapes on the hose base (b) Attach the lung compartment to the aluminium base, ensure the hole of the hose base is concentric with the hole on the top surface of the aluminium base (c) Side view of the assembly.

The construction of each lung compartment starts with assembling the bulkhead fitting and the elbow connector to the hose base, as depicted in Fig. 5. Next, the PVC aluminium ducting hose is cut to a length of 80 mm and adhesive is applied to the edges of its top and bottom surfaces, which are then attached to the hose cover and base, respectively, as visualised in Fig. 6. It is important to ensure the edges of the hose are properly sealed with adhesive to prevent any leakage. After the adhesive is cured, the lung compartment can be attached to the aluminium base with double-sided adhesive tape, as shown in Fig. 7.

**Fig. 8:**
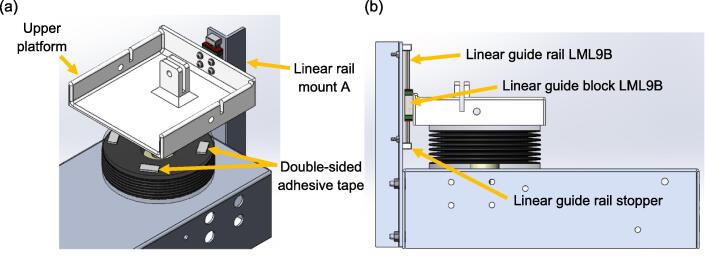
(a) Figure showing the double-sided adhesive tapes that hold the upper platform on the hose cover (b) The upper platform is attached to the hose cover of the lung compartment, where the linear guide rail stoppers are attached to the ends of the guide rail using adhesive to prevent the sliding block from going off the rail.

To ensure a smooth vertical motion during inflation or deflation of the lung compartment, a linear guide rail and block are used to guide the motion of the upper platform, which is attached to the hose cover of the lung compartment with double-sided adhesive tape. Referring to Fig. 8, the upper platform is secured on the linear guide block with four M3 x 8 mm bolts and washers. The linear guide rail is fastened to linear rail mount A with two M3 x 14 mm bolts and nylon insert lock nuts, along with two M3 washers. Linear rail mount A is then fastened to the aluminium base with two M6 x 16 mm bolts and nylon insert lock nuts, along with four M6 washers. A linear guide rail stopper is attached to each end of the linear guide rail with adhesive to prevent the sliding block from falling off the rail.

**Fig. 9:**
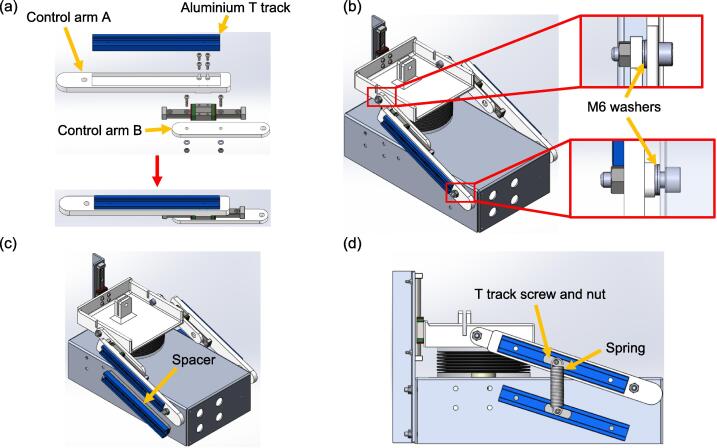
Steps to fabricate the spring-based mechanism (a) Assemble the control arm (the T track is attached to control arm A with adhesive) (b) Install the control arms on the test lung (c) The lower T track is attached to a spacer, which is then attached to the aluminium base with adhesive (d) Ensure the lower T track is parallel with the upper T track when the lung compartment is deflated, insert the T track nuts and screws, which will hold the spring.

The control arm assembly that is connected to the upper platform consists of an aluminium T track with a length of 150 mm, control arms A and B, a linear guide rail, a block, and two stoppers. Referring to Fig. 9(a), the linear guide rail is attached to control arm B with two M3 x 14 mm bolts, nuts, and washers. Four M3 x 8 mm bolts are then used to connect control arm A to the linear guide block. Lastly, the aluminium T track is fixed into the slot of control arm A with adhesive. The control arm assembly is attached to the upper platform and the aluminium base with a M6 x 20 mm and a M6 x 25 mm bolt, along with a nylon insert lock nut, respectively, as shown in Fig. 9(b). These connections act as pin supports, which allow rotational motion. Therefore, the fasteners should be tightened only until the components they hold are just touching each other. Fig. 9(c) and (d) show the lower aluminium T track, as well as the T track screws and nuts that hold the spring. While attaching the lower T track to the aluminium base, ensure the vertical distance between the two T tracks is the same as the unstretched length of the spring.

### 5.2 Respiratory airway

**Fig. 10:**
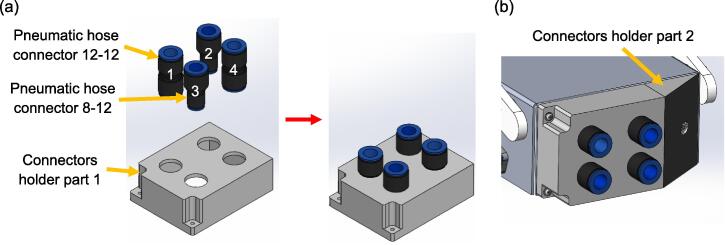
Steps to fabricate the inlets of the airways (a) The pneumatic hose connectors are fixed to the connectors holder part 1 with adhesive (b) Connectors holder part 2 is attached to part 1 with adhesive, the assembly is then fastened to the aluminium base.

After building the lung compartment, the next step will be fabricating the respiratory airway. Fig. 10 displays the procedures to fabricate the airway inlets, which are secured to the front of the aluminium base. These inlets are designated for different airway resistances. Among these inlets, inlets 1 and 4 utilise pneumatic hose connectors 12–12, whereas inlets 2 and 4 utilise connectors 8–12, where the connectors are fixed to the holder with adhesive. The holder is then fastened to the aluminium base with three M3 x 14 mm bolts and nuts, along with six M3 washers.

**Fig. 11:**
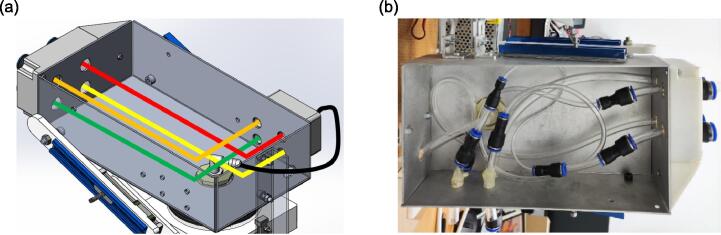
(a) There are four airways in the lung compartment, where the green, yellow, orange, and red airways are connected to connectors 1, 2, 3, and 4 respectively (b) The airways within the aluminium base.

**Fig. 12:**
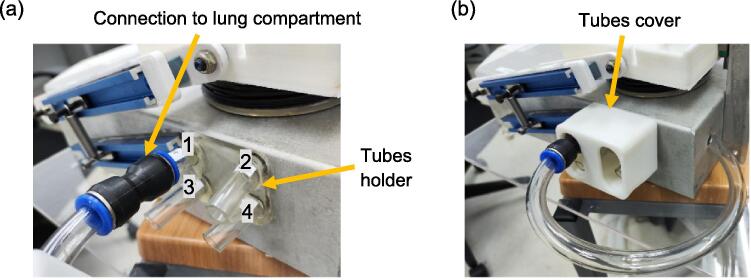
(a) The connection between the airway outlets and the lung compartment, the attachments between the tubes holder and the aluminium base, as well as between the tubes holder and the pneumatic tubes, are done by adhesive (b) 3D-printed tubes cover.

The simplified diagram and the actual setup of the respiratory airways are shown in Fig. 11(a) and (b), respectively. To achieve the desired airway resistance, each airway employs pneumatic tube(s) and connector(s) with different lengths and diameters, as summarised in Table 1. The respiratory airways should be fabricated following the sequence of pneumatic tube/connector listed in the table, where the first and last components should be connected to the inlets and outlets depicted in Fig. 10, Fig. 12, respectively.

**Table 1:** List of pneumatic tubes and connectors within the respiratory airways.

As mentioned previously, Fig. 12 displays the airway outlets located at the side of the aluminium base. The outlets are numbered corresponding to their inlet and are securely held in place by the tube’s holder. The connection between these outlets and the elbow connector of the lung compartment depicted in Fig. 5 is established through a pneumatic hose connector 12–12 and a 12 mm OD pneumatic tube, as outlined in Table 1. These outlet tubes are covered with a tubes cover. This step concludes the construction of a single lung compartment alongside the airways. As the final design comprises two separate lung compartments, a mirrored version of the assembly is needed to complete the mechanical test lung.

### 5.3 Spontaneous breathing system

**Fig. 13:**
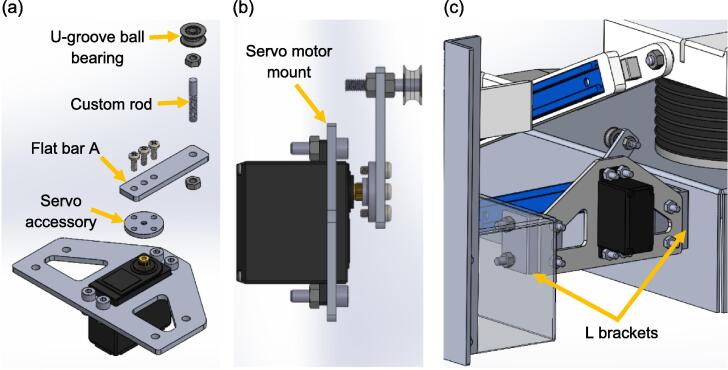
(a) Exploded view of the servo motor assembly (b) Side view of the servo motor assembly (c) Installation of the servo motor assembly to the test lung.

The next step in developing the product is to fabricate the spontaneous breathing system. Referring to Fig. 13(a) and (b), the U-groove ball bearing is pressed fit to the custom rod, which is held on flat bar A by two M4 nuts. Flat bar A and the servo accessory are then secured to the servo motor with three M3 x 8 mm bolts. Moving on, the servo motor is attached to the servo motor mount by four M4 x 16 mm bolts and nuts. The servo motor assembly is securely mounted to the test lung using two L brackets. This setup employs four M4 x 16 mm bolts and nuts to connect the servo motor mount to the L brackets, while four M5 x 16 mm bolts and nuts are used to attach the L brackets to the aluminium bases.

**Fig. 14:**
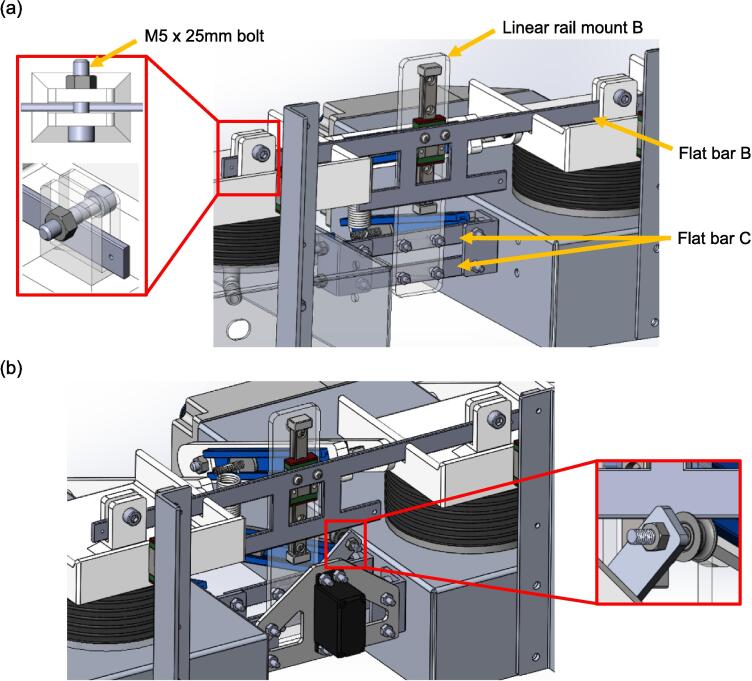
(a) Lifting arm assembly that control the vertical motion of the upper platforms of the lung compartments (b) Servo motor assembly and the accessory lifting arm.

Fig. 14(a) illustrates the lifting arm assembly components and their interconnections. The linear guide rail is affixed to linear rail mount B using two M3 x 14 mm bolts and nuts, accompanied by two M3 washers. Four M4 x 16 mm bolts and nuts secure two flat bar Cs to linear rail mount B, and these flat bar Cs are then mounted to the L brackets using four M4 x 16 mm bolts and nuts. The L brackets are attached to the aluminium bases with four M5 x 16 mm bolts and nuts. Finally, flat bar B is fastened to the linear guide block using two M3 x 8 mm bolts and washers. The connection enabling the lifting of the upper platforms is depicted in the highlighted region of Fig. 14(a). Fig. 14(b) shows the complete mechanism of the spontaneous breathing system for better visualisation.

**Fig. 15:**
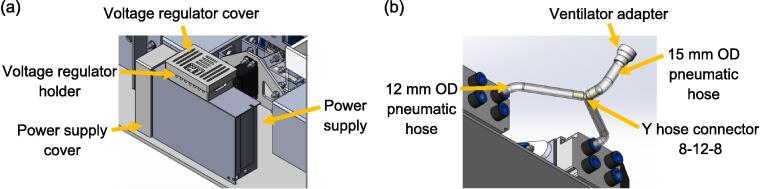
(a) Power supply for the spontaneous breathing system (b) Connecting hose between test lung and ventilator.

The power source of the spontaneous breathing system consists of a power supply and a voltage regulator, where the electrical wiring is shown in Fig. 4(c). The voltage regulator ensures compatibility with the servo motor's maximum voltage rating of 8.4 V by stepping down the 12 V voltage output from the power supply. To enhance safety, the electrical terminals are enclosed within the power supply cover, voltage regulator holder, and cover depicted in Fig. 15(a). In addition, both the power supply and the voltage regulator holder are securely positioned using double-sided tape. At the other end, the connection between the test lung and the ventilator is achieved through two 12 mm OD pneumatic hoses with a length of 285 mm, a Y hose connector, a 15 mm OD pneumatic hose with a length of 120 mm, and a ventilator adapter, as shown in Fig. 15(b).

### 5.4 Calibration

**Fig. 16:**
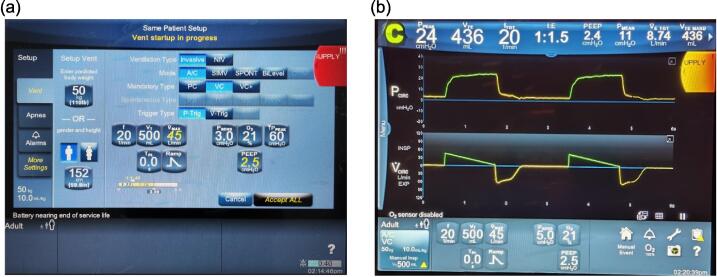
(a) Settings of PB980 ventilator during calibration of the test lung (b) Data displayed on PB980 ventilator during the ventilation.

**Fig. 17:**
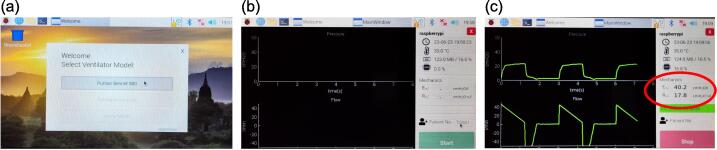
Steps to activate CAREDAQ for calibration of the test lung (a) Select PB980 as the ventilator model (b) Define patient no. after launching the DAQ software (c) Start the data acquisition, the lung elastance and airway resistance are shown in the area circled in red.

The final step concluding the fabrication process is to calibrate the lung elastance and airway resistance of the test lung. Calibration is achieved by connecting the test lung to a Puritan Bennett 980 (PB980) or similar standard of care ventilator, with the ventilator settings showcased in Fig. 16(a). Once the ventilator is configured to the desired settings, ventilation is initiated by clicking on the “Accept ALL” button, and subsequent ventilation data is displayed, as depicted in Fig. 16(b). These data would be retrieved by a data acquisition device, which in this case was the CAREDAQ system [13]. However, any similar acquisition system and sensors would suffice if sufficiently accurate E.g. [12], [14]. Fig. 17 summarises the steps used here to activate CAREDAQ for calibration purposes. CAREDAQ calculates the mechanical test lung elastance and airway resistance by performing linear regression to the airway pressure and flow measurements fitting to a single compartment model [8]. Constant elastance and resistance of the single compartment model are reasonable approximations to what is observed over a MV breath, and thus, it is used to calibrate the mechanical test lung.

## 6 Operation instructions

**Fig. 18:**
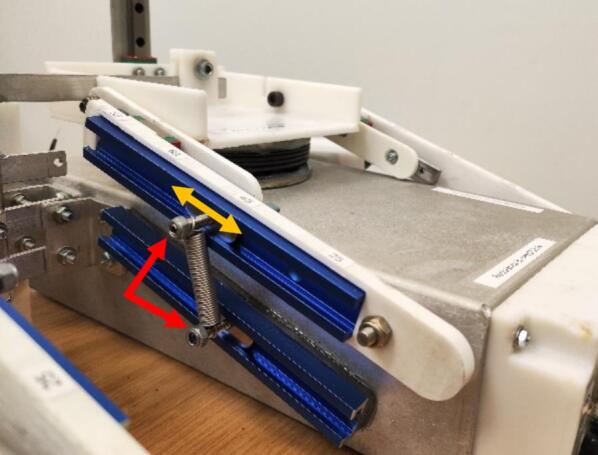
The studs with nylon lock nuts at the end (pointed in red) need to be loosened to allow the spring to slide along the aluminium track.

**Fig. 19:**
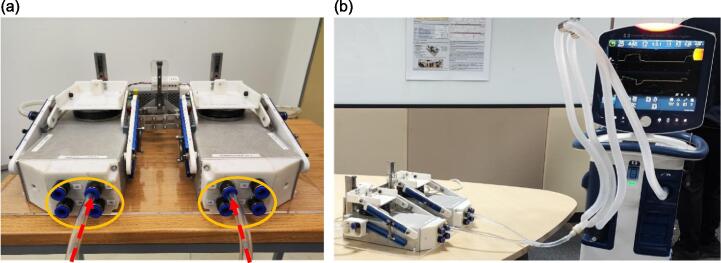
(a) Insert each pneumatic tube into the connector with the desired airway resistance (b) Connection between the test lung and the ventilator.

Before using the test lung, the user must select the respiratory mechanics, which are the lung elastance and airway resistance to be simulated. Referring to Fig. 18, the lung elastance can be adjusted by moving the spring along the aluminium track to the designated calibrated position. This adjustment needs to be done on all four springs of the test lung to achieve the desired lung elastance. Meanwhile, the required airway resistance could be achieved by inserting the pneumatic tubes into the corresponding connectors, as depicted in Fig. 19(a). Once configured to the correct settings, the test lung is ready for connection to the ventilator, as demonstrated in Fig. 19(b).

**Fig. 20:**
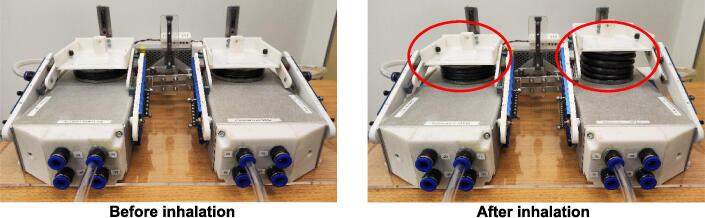
Independent lung compartments with standalone mechanism for the adjustment of airway resistance and lung elastance. After inhalation, the right compartment with lower elastance receives higher air volume compared to the compartment with higher elastance.

The test lung consists of two independent lung compartments, with each having a standalone mechanism for the adjustment of lung elastance and airway resistance. Therefore, each compartment can be set with different combinations of these two parameters to simulate lung heterogeneity to a certain extent during passive ventilation, as shown in Fig. 20 below. The heterogeneous lung elastance can be simulated by setting different spring positions for each compartment and detaching the Flat bar B shown in Fig. 14(a).

**Fig. 21:**
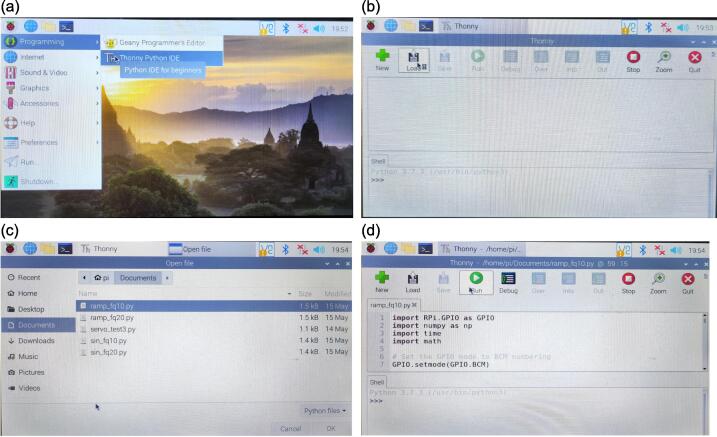
Steps to activate the spontaneous breathing system (a) Launch the Thonny Python IDE (b) Open the folder with the provided codes (c) Select the desired breathing waveform and frequency (d) Run the code.

To activate the spontaneous breathing system, power up the RPi and the servo motor using their respective power supplies: the RPi power supply for the RPi and the S-120–12 power supply for the servo motor. After switching on the RPi, initiate Thonny by navigating through the RPi icon, then selecting Programming, and finally Thonny Python IDE. Within the Thonny interface, the provided codes allow for the generation of two distinct waveforms, sinusoidal and triangular, each offering two breathing frequencies: 10 and 20 breaths per minute. Following the selection of the preferred breathing waveform and frequency, load the corresponding file in Thonny and click the Run button. Fig. 21 provides visualisation of the mentioned steps.

## 7 Validation and characterization

**Fig. 22:**
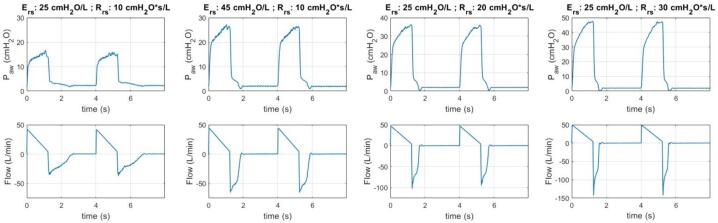
Ventilation data for different lung elastances.

**Fig. 23:**
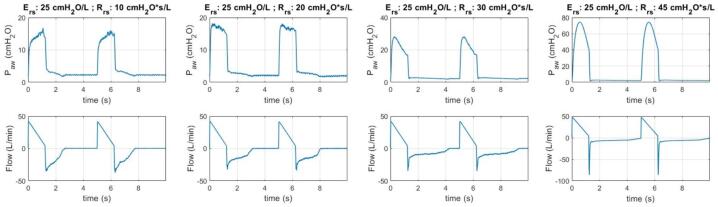
Ventilation data for different airway resistances.

**Fig. 24:**
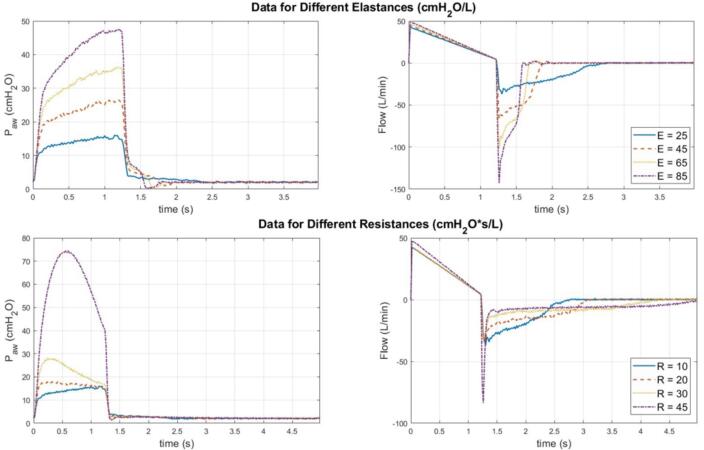
Comparison of ventilation data under different settings of lung mechanics.

This section presents the outcomes of the experiment regarding the ventilation of the CARE Test Lung under various lung mechanics settings. Fig. 22, Fig. 23 illustrate the ventilation data of the test lung under different levels of elastance and resistance, respectively. These figures demonstrate the consistent, replicable performance of the test lung across multiple breathing cycles. In contrast, Fig. 24 displays the data for a single cycle under each of these lung mechanics settings, offering a more detailed and easily understandable comparison of ventilation patterns among these configurations.

Based on the ventilation data for different lung elastances in Fig. 24, it is evident a higher lung elastance correlates with increased airway pressure and expiration flow rate, as demonstrated by the pressure–time and flow-time graphs, respectively. Conversely, when examining the data for different resistances, it becomes apparent higher airway resistance leads to increased airway pressure during early inspiration, but a reduced expiration rate. All these behaviours are expected [15], [16].

**Fig. 25:**
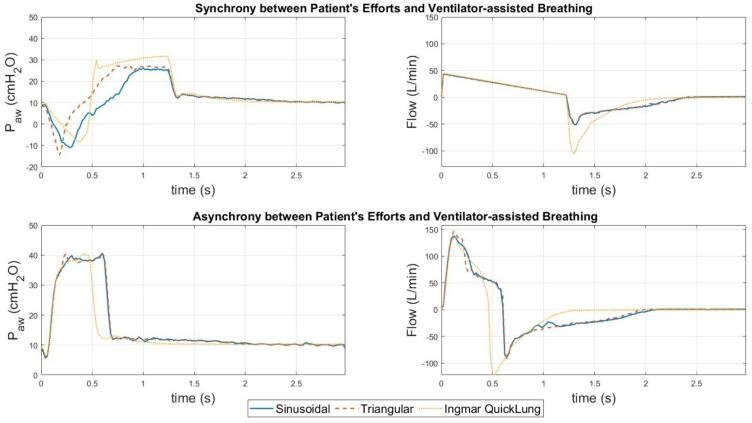
Comparison of ventilation data with different spontaneous breathing waveforms.

Fig. 25 presents the ventilation data when spontaneous breathing efforts are initiated by the test lung. In the pressure–time graph of synchronous breathing, the trough generated by the triangular waveform is narrower than that of the sinusoidal waveform. However, both waveforms show identical flow data, as depicted in the flow-time graph. Comparing synchronous and asynchronous breathing, asynchronous breathing exhibits around a 40 % higher peak airway pressure (peak inspiration pressure) and a maximum flow rate three times higher than synchronous breathing. For validation purposes, the ventilation data of the Ingmar QuickLung is included for comparison. Based on the figure, it could be seen that the expiration waveforms of the test lung deviate from that of the QuickLung. This difference is primarily caused by the distinct spring-loaded mechanisms used by the two systems to replicate lung elastance. The QuickLung system, with its straightforward design, provides a smoother expiratory cycle. Nevertheless, the test lung presented is more versatile as it offers a continuous range of lung elastance settings.

In evaluating the device's performance, a comparison was made with several established clinical test lungs. Based on the specifications listed in Table 2, CARE Test Lung not only possesses the most options for the resistance settings but also gives a continuous range of elastance, providing the highest versatility in simulating different patients among the listed models.

**Table 2:** Specifications of different test lung models.

	CARE Test Lung	Ingmar QuickLung	Vadi Test Lung
Elastance (cmH_2_O/L)	25 to 85 (continuous)	20, 50, 100	45
Resistance (cmH_2_O·s/L)	10, 20, 30, 45	5, 20, 50	10
Type	Active	Active	Passive

In summary, this paper covers the building and operation instructions of a mechanical test lung that can simulate the diverse physiological responses of patients with respiratory failure, particularly those with acute respiratory distress syndrome (ARDS), during MV treatment. While the proposed mechanical test lung is similar to commercial devices, commercial devices are not open designs that anyone can build off of. Its cost-effectiveness and comprehensive documentation make this test lung a valuable resource for advancing education in respiratory mechanics research and clinical training, particularly in resource-limited developing countries.

## CRediT authorship contribution statement

**Wei Yang Tay:** Writing – review & editing, Writing – original draft, Validation, Software, Methodology, Formal analysis, Data curation, Conceptualization. **Christopher Yew Shuen Ang:** Writing – review & editing, Writing – original draft, Validation, Methodology, Investigation, Formal analysis. **Yeong Shiong Chiew:** Writing – review & editing, Writing – original draft, Supervision, Resources, Project administration, Methodology, Conceptualization. **J. Geoffrey Chase:** Writing – review & editing, Funding acquisition.

## Declaration of competing interest

The authors declare that they have no known competing financial interests or personal relationships that could have appeared to influence the work reported in this paper.
